# Beyond tumor-associated macrophages involved in spheroid formation and dissemination: Novel insights for ovarian cancer therapy (Review)

**DOI:** 10.3892/ijo.2024.5705

**Published:** 2024-11-07

**Authors:** Yuchen Liu, Haoyue Xiao, Hai Zeng, Ying Xiang

**Affiliations:** 1 Laboratory of Oncology, Center for Molecular Medicine, School of Basic Medicine, Health Science Center, Yangtze University, Jingzhou, Hubei 434023, P.R. China; 2 Department of Oncology, First Affiliated Hospital of Yangtze University, Jingzhou, Hubei 434023, P.R. China; 3 Department of Cell Biology and Medical Genetics, School of Basic Medicine, Health Science Center, Yangtze University, Jingzhou, Hubei 434023, P.R. China

**Keywords:** ovarian cancer, tumor-associated macrophage, spheroid, peritoneal metastasis

## Abstract

Ovarian cancer (OC) is the most common and deadly malignant tumor of the female reproductive system. When OC cells detach from the primary tumor and enter the ascitic microenvironment, they are present as individual cells or multicellular spheroids in ascites. These spheroids, composed of cancer and non-malignant cells, are metastatic units and play a crucial role in the progression of OC. However, little is known about the mechanism of spheroid formation and dissemination. Tumor-associated macrophages (TAMs) in the center of spheroids are key in spheroid formation and metastasis and provide a potential target for OC therapy. The present review summarizes the key biological features of spheroids, focusing on the role of TAMs in spheroid formation, survival and peritoneal metastasis, and the strategies targeting TAMs to provide new insights in treating OC.

## 1. Introduction

Ovarian cancer (OC) has the highest mortality rate (>314,000 new cases and around 207,000 new deaths worldwide in 2020) among all gynecological malignancies in the world due to lack of symptoms and effective makers at early stages (1). If the cancer is restricted to the ovaries (stage I), up to 90% of patients can be treated with currently available treatments (surgery, chemotherapy, anti-angiogenic therapy, immunotherapy). Even if the disease has spread to the pelvic organs (stage II), up to 70% of patients survive for >10 years. However, if it further disseminates into the peritoneum or the surface of abdominal organs (stage III) or outside the abdomen (stage IV), 5-year survival rate declines to ≤20% (2). For most patients with OC, tumor cytoreduction surgery is their last option at the advanced stage with intraperitoneal and extensive pelvic implantation metastasis. Even with aggressive first-line chemotherapy following optimal debulking surgery, the initial cure rate is 80%. The majority of patients with advanced-stage OC exhibit unsatisfactory response due to acquired chemoresistance, which results in recurrence and chemotherapy failure (3).

Increase in the amount of fluid in the abdominal cavity >200 ml is termed ascites. Ascites formation serves a vital role in the progression of OC, serving as a transporter of tumor cells from the primary location to metastatic sites (4). The incidence of ascites varies between the four stages of OC, ranging from 49.4 at stage I to 62.5 in stage II and 90.1 and 100.0% in stages III and IV, respectively (5). Malignant ascites (MA) contains cellular and acellular components. Cellular components include cancer, immune and mesothelial cells and fibroblasts, while acellular components include proteins, such as cytokines and growth factors, metabolites and exosomes (6). Massive ascites may cause abdominal distension, respiratory compromise, anorexia and cachexia (7). Decreased lymphatic absorption and increased fluid production via high vascular permeability are the primary factors contributing to MA formation. To date, a gold standard for clinical management of MA has not been clearly defined. Paracentesis and diuretics relieve the accumulation of ascites, but their efficacy is often partial and temporary (8).

Unlike other solid cancers, OC rarely exhibits hematogenous and lymphatic metastasis. Peritoneal (trancoelomic) metastasis is more frequent and can be detected in ~70% of OC cases (9). Tumor cells implant directly into adjacent organs after detachment from the primary site or spread to the omentum, parietal and visceral peritoneum via peritoneal fluid or ascites. In OC ascites, cancer cells float as individual cells or multicellular aggregates (also called spheroids). These malignant cells accumulate as globular structures, thus resisting anoikis and helping to spread throughout the abdominal cavity (10). Spheroids, exerting higher tumorigenic and chemoresistant properties than individual cancer cells (11), are considered metastatic units of peritoneal dissemination (12). Beside tumor cells, OC ascites also contains non-tumor cellular components, such as macrophages, lymphocytes, fibroblasts, adipocytes and mesothelial cells (13). The interaction of tumor and non-tumor cells in ascites leads to formation of heterogeneous spheroids. Such heterospheroids are more invasive and more resistant to anoikis and chemotherapeutic drugs than homospheroids composed of cancer cells alone (14).

Tumor-associated macrophages (TAMs) are the most frequent cell type (up to 50% of the total) in the ascitic microenvironment (15). Recently (16), it has been revealed that TAMs exist in the center of spheroids, regulating spheroid formation, survival and adhesion to peritoneum and then regulating peritoneal metastasis. TAMs are key for OC progression (17). Thus, it is of importance to study the role and molecular mechanisms of TAMs in spheroid formation and intraperitoneal implantation (18). Compared with previous reviews, which primarily focused on the immunosuppressive role of TAMs in cancer (15,19,20) or only briefly mentioned TAMs in OC spheroids (21-23), the present review investigates the role of TAMs in spheroid formation and dissemination in OC progression, the molecular mechanisms by which TAMs participate in peritoneal metastasis of OC, including spheroid formation, survival and dissemination, as well as strategies targeting TAMs in OC in clinical or preclinical research.

## 2. Role of spheroids in progression of OC

**Figure 1:**
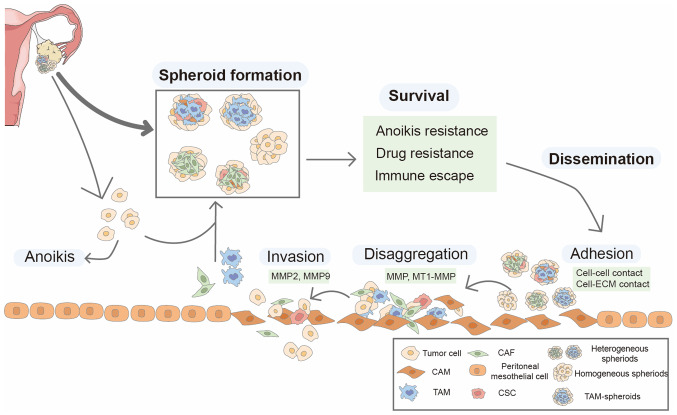
Role of spheroids in the progression of ovarian cancer. Spheroids floating in ascites include homogenous spheroids and heterogenous spheroids. Homogenous spheroids are composed of cancer cells only, while heterogenous spheroids are composed of cancer and other stromal cells, such as TAMs and CAFs. These spheroids primarily detach from the primary tumor; few are formed by aggregation of individual stromal cells and cancer cells. Compared with individual cells, multicellular spheroids are more likely to survival in ascites as they exhibit anoikis and chemotherapeutic drug resistance and immune escape. Spheroids adhere to the peritoneum covering by a single layer of mesothelium cells with cell-cell interactions or cell-ECM interactions. These mesothelial cells are induced to CAMs by cancer cells. Once adhered, spheroids disaggregate on ECM and invade the ECM by activating MMP2/MMP9 and MT1-MMP. TAM, tumor-associated macrophages; CAF, cancer-associated fibroblast; ECM, extracellular matrix; CAM, cancer-associated mesothelial cell; CSC, cancer stem cell; MT1, melatonin receptor type 1.

OC cells metastasize directly from the primary site to the abdominal cavity, where they survive and travel as individual cells or multicellular aggregates in the peritoneal fluid or ascites, then adhere to peritoneal tissue, anchor to the submesothelial matrix, and proliferate to form secondary lesions (24). These spheroids exist in, and can be isolated, from OC ascites. The role of spheroids in OC progression is summarized in Fig. 1.

### Formation of spheroids in ascites

Spheroids contain cancer and non-cancer cells such as cancer-associated fibroblasts (CAFs), TAMs and rare cancer stem cells (CSCs). Spheroids have different sizes and structures (25). Spheroids may be formed by aggregation of individual stromal and cancer cells in ascites or clusters detached from the primary tumor. In mice, intraperitoneal aggregation is not the main mechanism of spheroid formation and 80% of OC spheroids are produced by clustered cells separating from the primary tumor, while intraperitoneal aggregative cells account for only a small fraction (10).

Three-dimensional (3D) cell culture is used for spheroids research *in vitro*. Compared with traditional monolayer cell culture, 3D spherical cells mimic the differentiation pattern *in vitro* and spatial contact with the intercellular and extracellular matrix (ECM) (26). The initial formation of spheroids begins with formation of relatively loose cell aggregates from integrin-ECM components, followed by expression of adhesion molecule E-cadherin, which binds to hemophilic cadherin-cadherin to form dense spheroids (27). E-cadherin has an important regulatory role in formation of tumor spheroids. Higher expression of E-cadherin is closely associated with compact spheroids (28). Mechanistically, decreased E-cadherin upregulates α5-integrin expression through epidermal growth factor receptor (EGFR)/focal adhesion kinase (FAK)/extracellular signal-regulated kinase 1 (ERK1) signaling, facilitating OC cell aggregation (29). NIH:OVCAR5 spheroids of OC cells in a 3D cell culture model of multicellular aggregates isolated from ascites show that α5β1 integrin and its ligand fibronectin are exposed on OC spheroids. Monoclonal antibodies against α5- or β1-integrin inhibit the formation of spheroids, suggesting that the interaction between α5β1-integrin and fibronectin serves a vital role in the aggregation of OC cells (30). In another study, ascitic tumor cells with high α5 integrin expression were selectively recruited by CAFs to form heterotypic spheroids (14). Moreover, EGF derived from CAFs under ascitic tumor cell stimulation is significantly enriched within heterotypic spheroids, where it increases integrin α5 expression on ascitic tumor cells, thereby strengthening interactions between ascitic tumor cells and CAFs. CAF-tumor cell spheroids are considered to be the metastatic units of high grade serous OC (14). Han *et al* (31) demonstrated that CAFs serve as a scaffold to gather floating tumor cells, promoting peritoneal metastasis by forming heterotypic aggregates with tumor cells. Upregulation of CD44 on the surface of OC cells is key during the progression of OC, since CD44 is a receptor for hyaluronate, a key component in ECM, which serves an important role in cell communication and adhesion between cells and the ECM. Inhibition of CD44 by its specific short hairpin RNA in OC cells decreases proliferation and spheroid formation (32).

Numerous signaling pathways are involved in spheroid formation. Chen *et al* (33) found that activation of signal transducer and activator of transcription 3 (STAT3) signaling pathway is associated with the formation of spheroids. In a mouse model of human ovarian carcinoma, STAT3 regulated formation of spheroids and self-renewal, while attenuation of STAT3 decreased the tumorigenicity. Wnt/β-catenin signaling is a key pathway in the regulation of the formation of spheroids through STAT3 (33). β-catenin is a marker for CSCs, which exist in the center of spheroids (34,35), and activation of β-catenin regulates the ability to initiate tumors and the formation of spheroids (36). Myeloid-derived suppressor cells enhance stemness of cancer cells, spheroid formation and cancer metastasis in an OC mouse model *in vivo* and microRNA (miR)-101-co-repressor gene C-terminal binding protein-2-SC core genes are involved in these effects (37). Furthermore, angiotensin II receptor contributed to the development and metastasis of OC (38). Angiotensin II significantly promotes the spheroid formation, growth and invasiveness of several OC cell lines due to the direct activation of the MAPK/ERK pathway and transactivation of EGFR), which upregulates the expression of stearoyl-CoA desaturase 1 gene, alters lipid metabolic homeostasis and inhibits endoplasmic reticulum stress within the spheroids (39).

### Survival of spheroids in ascites

#### Resistance to anoikis

Anoikis is a specific form of apoptosis due to insufficient or poor cellular adhesion (40). Anoikis is regulated by integrins, which interact with ECM components to form adhesion complexes (41). Either through intrinsic or extrinsic apoptotic pathways, anoikis prevents epithelial cells from detaching from their original location and colonizing new sites (42). Tumor cells are less sensitive to anoikis than normal epithelial cells. Cancer cells downregulate intercellular adhesion molecules via epithelial-mesenchymal transition (EMT), inhibiting E-cadherin expression to decrease cadherin-dependent intercellular contact and allow cancer cells to resist anoikis (43). Cancer cells develop several mechanisms for abrogating anoikis, such as activating Src/AKT/ERK signaling, which is involved in anoikis resistance via blocking the mitochondrial pathway and glycolysis (44). The Notch signaling pathway, which is initiated by receptor (Notch1-4)-ligand (Δ and Jagged) interaction, has been considered a potential therapeutic target for OC (45). High expression of Notch3 is associated with poor prognosis in epithelial OC (46). Elevated Notch3 expression promotes anoikis resistance via upregulation of type IV α2 collagen (COL4A2) gene, a key component of the basement membrane that allows OC cells to maintain survival-friendly signaling by spoofing proteins responsible for detecting ECM contact, such as integrins, without making contact with ECM; FAK/AKT/ERK1/2 activation is the key mechanism (47). Hepatocyte growth factor (HGF) receptor c-Met is frequently highly expressed in OC and contributes to anoikis resistance. The effects are dependent on both phosphatidylinositol 3-kinase (PI3K)/AKT and ERK1/2 signaling pathways and Ras serves as a central role for the cross talk (48). In addition, cancer cells commonly evade apoptosis by upregulating anti-apoptotic Bcl-2 family proteins and/or downregulating pro-apoptotic proteins (49). Frizzled family receptor 7 (FZD7), which mediates both classical and non-classical Wnt signaling, plays an important role in maintaining SC properties as well as tumor development (50). A study (51) demonstrated the regulatory role of FZD7 on spheroid proliferation of CSCs in OC via activation of the Wnt/β-catenin pathway. TWIST1 is an important regulatory molecule of the Wnt3a/Wnt1/β-catenin signaling pathway, which is closely related to mesenchymal and tumor stem cell phenotypes (52). Tan *et al* (53) found that the FZD7/TWIST1/Bcl-2 signaling pathway played a role in the maintenance of mesenchymal phenotype and anoikis resistance and was involved in OC spheroid formation. FZD7 promoted TWIST1 expression via epigenetic modifications of H3K4me3 and H3K27ac at the TWIST1 proximal promoter; TWIST1 regulated the expression of Bcl-2, an anti-apoptotic protein (53).

In ascites, individually suspended tumor cells are more prone to anoikis than clustered cells. Multicellular spheroids consisting of tumor cells surrounded by immune and stromal cells show enhanced survival compared with individual tumor cells (54). Long non-coding RNA HOTAIR is a key indicator of poor prognosis in patients with OC. Dai *et al* (55) found that HOTAIR is upregulated in OC cells in suspension culture and allows cells to acquire anoikis resistance. Silencing of HOTAIR in SKOV3 cells inhibits spheroid formation, decreases aggressiveness and enhances chemosensitivity. HOTAIR promotes enhancer of zeste homolog 2 (EZH2) expression; EZH2-mediated methylation of lysine 27 on histone H3 (H3K27) contribute to the formation of spheroids (55). HOTAIR can also serve as a competitive endogenous RNA to regulate phosphoinositide-3-kinase regulatory subunit 3 (PIK3R3) and promotes proliferation, migration and invasion of OC cells. PIK3R3 is a subunit of PI3K and activation of the PI3K/AKT signaling pathway is key for cell survival (56,57). Activation of caspase-3 is a common event in both intrinsic and extrinsic anoikis. In spherical OC cells, AKT kinase is activated and promotes OC survival through inhibiting caspase-3 (58). Tropomysin-related kinase B (TrkB), a neurotrophic tyrosine kinase receptor, is overexpressed in OC tissue, particularly in greater omentum metastatic lesions and multicellular spheroids in ascites. TrkB mediates suppression of anoikis via activating the PI3K/AKT pathway (59). Furthermore, clustered cancer cells express specific adhesion molecule αv-integrin to activate survival signaling pathways. αv-integrin maintains cell survival via ERK1/2 activation, thereby enhancing the resistance of OC tumor spheroids to anoikis (60). Moreover, there are genetic differences between tumor cells in primary tissue and ascites. After tumor cells detach from primary tissue and disseminate into the abdominal cavity, tumor cells undergo independent clonal evolution. KRAS mutation in ascites leads to acquired anoikis resistance, which increases the survival of tumor cells in ascites (61).

### Resistance to chemotherapeutic drugs

The spheroids in OC ascites vary in shape and size (62). It was showed that spheroids could inhibit the entry of chemotherapeutic drugs into cells to inhibit their therapeutic effects (63). The mechanism is associated a number of factors such as the 3D spatial structure inside spheroids, drug gradient penetration, cell-cell contact and low pH, dense or loose structure of spheroids and the mixed cell types (necrotic, quiescent or proliferating cells) (64). Cell adhesion molecules are key in the formation of spheroids (65). Cell division cycle 25 A (CDC25A) is a phosphatase that regulates cell cycle progression through the G1/S and G2/M checkpoints (66). CDC25A is highly expressed in patients with OC (67). CDC25A maintains the structural stability of multicellular tumor spheroids by upregulating the expression of E-cadherin protein, which improves the tolerance of OC spheroids to chemotherapeutic resistance (65). Green *et al* (68) showed that in HT29 multicellular spheroids cultured *in vitro*, E-cadherin-mediated adhesion is sensitive to 5-fluorouracil, paclitaxel, vincristine and etoposide, but not to cisplatin, after disturbance of adhesion function.

Numerous proteins involved in drug resistance are overexpressed in spheroids (69). One study compared the drug sensitivity of spherical OVAR-3 and OVAR-8 cell lines in 3D culture system with monolayer cell lines in 2D culture system: Spheroids were more resistant to cisplatin and taxol compared with monolayer of OC cell lines, and spheroids took up less taxol than monolayer cells. Large spheroids were more resistant to taxol. However, the spheroids were equally sensitive to cisplatin and there was no significant difference based on size and morphology (70). The different responses of spheroids to taxol and cisplatin may be due to the different mechanisms of these drugs: Taxol inhibits cell division by suppressing dynamics of microtubules and proper assembling of mitotic spindle; while cisplatin causes the breakage of DNA strands by formation of covalent adducts between platinum complexes and DNA (71,72).

A large number of mesothelial cells exist in spheroids (27), which may contribute to resistance to drug-induced apoptosis via releasing pro-survival factors, which activate AKT and NF-κB survival pathways. In addition, CSCs also exist in spheroids. CSCs are key for tumorigenesis, chemoresistance and recurrence. Aldehyde dehydrogenase 1 (ALDH1) is important in study of the relationship between expression and poor prognosis in ovarian tumor SCs (34). Increased ALDH activity is found in spheroids (35). CSCs are the primary cause of high recurrence rates of OC (73). When chemotherapeutic drugs are present, non-SCs in spheroids are killed by drugs but stem-like cells survive. Spherical OC cells overexpress stem cell genes under long-term treatment with chemotherapeutic drugs (74). CSC maintenance is dependent on Notch signaling pathway, especially Notch3, which also serves a key role in platinum chemoresistance (76). Inhibition of Notch3 by small interfering RNA markedly decreases the size and number of spheroids (76).

**Figure 2:**
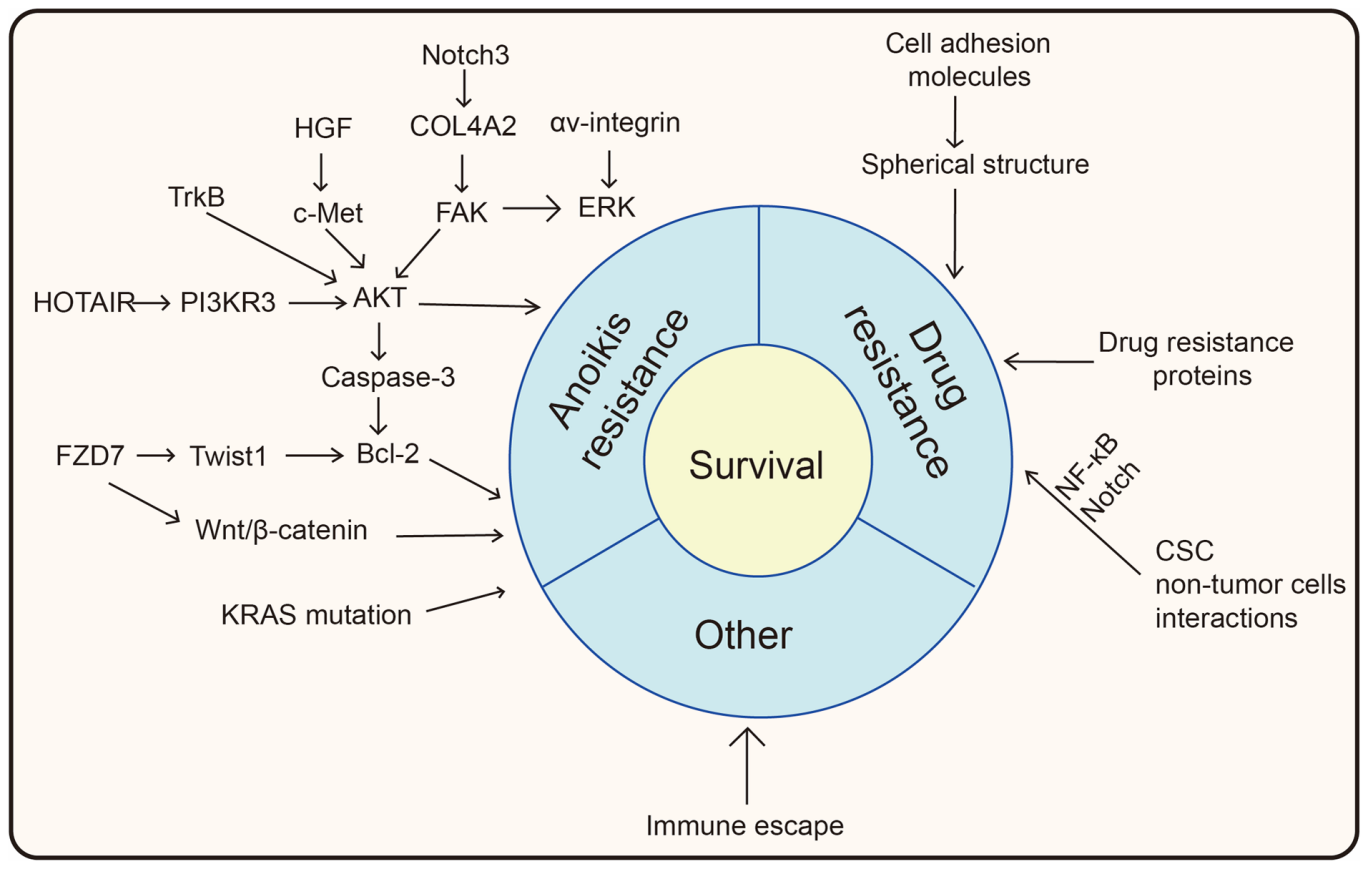
Survival mechanisms of ovarian cancer spheroids in ascites. Several signaling pathway are involved in anoikis resistance, such as AKT pathway, Bcl-2 pathway, and Wnt/β-catenin pathway. Resistance to chemotherapeutic drugs may be due to spherical structure, drug resistance-related proteins, CSCs and other non-tumor cells present in spheroids. Other mechanisms such as immune escape may also contribute to survival of spheroids. FZD7, Frizzled family receptor 7; PIK3R3, phosphoinositide-3-kinase regulatory subunit 3; COL4A2, type IV α2 collagen; TrkB, tropomysin related kinase B; HGF, hepatocyte growth factor; ERK, extracellular signal-regulated kinase; FAK, focal adhesion kinase; CSC, cancer stem cell.

The mechanisms by which spheroids acquire resistance to anoikis and chemotherapeutic drugs are summarized in Fig. 2. After acquiring anoikis and chemotherapeutic resistance, as well as immune escape in the immunosuppressive ascitic microenvironment, spheroids may survive in the ascites, and then disseminate to the peritoneum.

### Peritoneal dissemination of spheroids

The peritoneum, the largest serous membrane of the human body, covering the abdominal and pelvic cavities and visceral organs, is a preferred location for trancoelomic metastasis of numerous types of epithelial malignancy, including ovarian, colonic and gastric cancer (77). The peritoneum is composed of a layer of mesothelial cells and associated underlying ECM. These mesothelial cells serve as initial barriers for cancer cells. However, they can be induced to cancer-associated mesothelial cells (CAMs) by cancer cells. Mesothelial cells undergo to mesothelial-mesenchymal transition induced by HGF secreted by OC cells, then CAMs promote the expression of pro-tumor factors such as IL-8 and C-X-C motif chemokine ligand 5 (CXCL5) to facilitate dissemination of OC cells (78). The greater omentum is the most common metastatic site of OC, since it lacks basement membrane and mesothelial cells on the surface of milky spots (79,80).

Mesothelial cells retract during peritoneal metastasis. Unlike normal peritoneal mesothelial cells, which are flattened and spread over the entire surface of the peritoneal cavity, the mesothelial cells are rounded and separated from each other during peritoneal metastasis, exposing the submesothelial surface. OC spheroids detach from the primary tumor and disseminate to the peritoneum though ascites. These spheroids adhere to the mesothelial cells through adhesion molecules such as CD44, α5β1, αvβ1 and α2β1 integrins and mesothelial cells undergo localized retracement and detachment (81). E-cadherin loss is often associated with metastasis of OC. In OC cells, inhibited E-cadherin expression significantly upregulates the expression of α5-integrin, a subunit of fibronectin receptor α5β1-integrin, which binds with fibronectin, mediating adhesion to the peritoneal ECM (29). Once the spheroids spread over the monolayer of mesothelial cells, mesothelial cells move out from directly beneath the spreading spheroids. This is termed mesothelial clearance (82). Spheroid-induced mesothelial clearance depends on α5β1 integrin, talin I and myosin II. Following binding of mesothelial cells, spherical cancer cells utilize integrin- and talin-dependent myosin activation and traction, promoting mesothelial cells to migrate from beneath the spheroids (82).

Mesothelial clearance leads to exposure of underlying ECM and promotes further attachment of cancer cells. Cancer cells express CD44 on the cell membrane, which binds to hyaluronan in ECM to strengthen the link with peritoneal mesothelium (83), and express integrins that bind the basement membrane composed of laminin, fibronectin and types I and IV collagen (84). Spheroids readily adhere to and disaggregate from ECM substrates, particularly fibronectin and collagen I (85). Disaggregation of spheroids into individual cells is necessary for invasion of the mesothelium (86). α2β1 integrin serves a vital role in the dissemination of ovarian carcinoma spheroids: α2β1 integrin on cancer cells adheres to type I collagen, followed by secretion of serine and metalloproteinases, and contributes to the metastasis of OC into the abdominal cavity (87). In an *in vitro* spreading homozygous spheroid model of OC, α2β1 integrin was upregulated in spheroids and associated with disaggregation from ECM and invasion by activating MMPs such as MMP2/MMP9 (88). Blockade of α2β1 integrin using monoclonal antibodies decreases disaggregation and proteolysis of spheroids (88).

## 3. TAMs in spheroid formation, survival and metastasis

**Figure 3:**
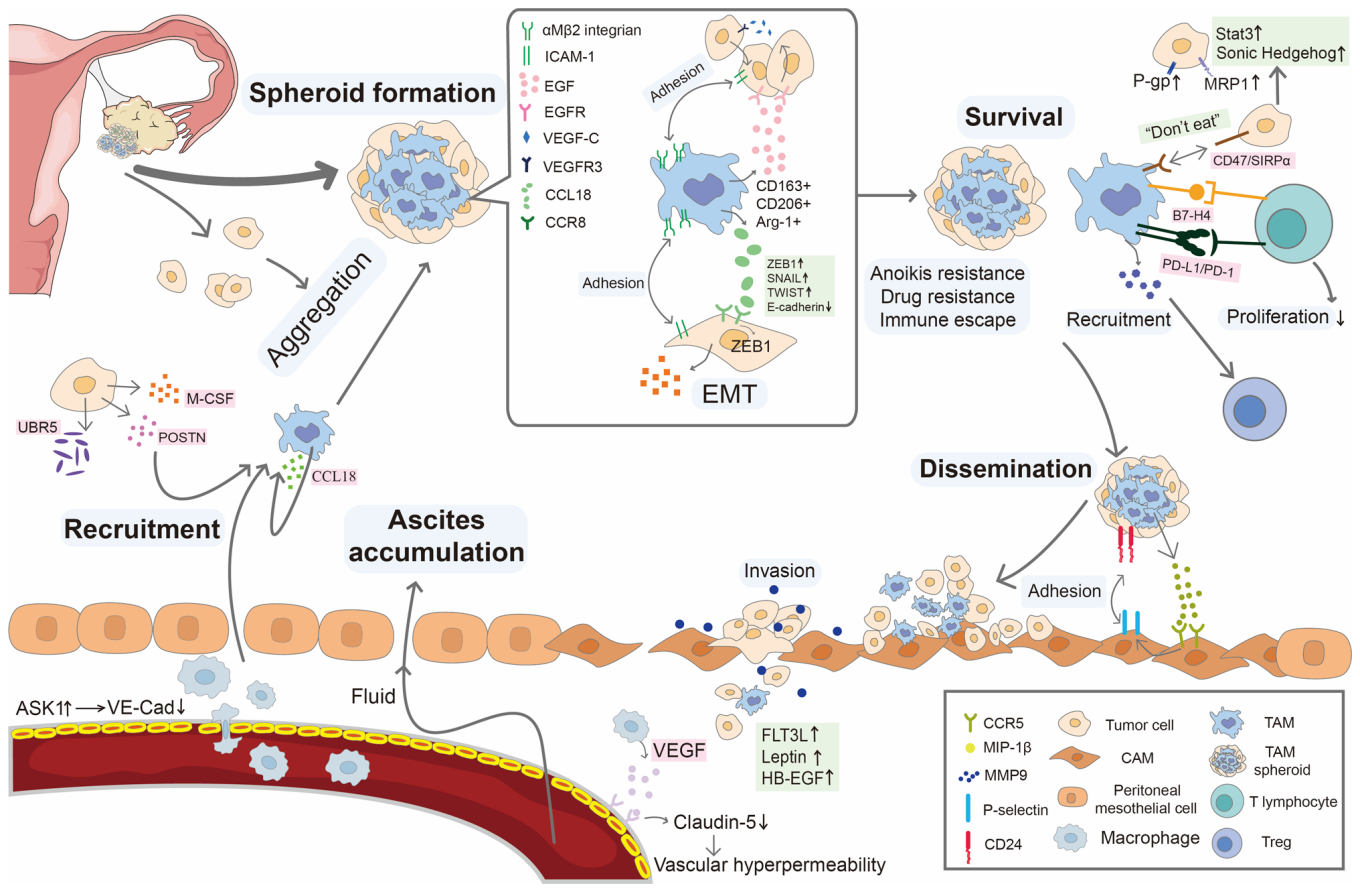
Role of TAMs in spheroid formation, survival and metastasis. TAM-secreted VEGF binds to its receptor KDR/Flk-1 on endothelial cells, then decrease expression of junctional protein claudin-5 on endothelial cells, increase vascular permeability and contributes to ascites accumulation. Macrophages are recruited to ascites by soluble factors released by cancer cells including UBR5, M-CSF and POSTN, and chemokines released by TAMs, such as CCL18. TAM spheroids in ascites primarily detach from the primary tumor. TAM-derived EGF activates EGFR on cancer cells and increases expression of VEGF-C and VEGFR3 in cancer cells. EGF also increases the expression of αMβ2 integrin in TAMs and ICAM-1 in cancer cells to facilitate adhesion between TAMs and cancer cells. TAM-released chemokine CCL18 interacts with CCR8 to promote EMT of cancer cells. TAMs promote anoikis and drug resistance via the activation of STAT3 and Sonic hedgehog pathways and upregulation of P-gp and MRP1 in cancer cells and promote immune escape via suppressed phagocytosis of TAMs via SIRPα, inhibit T cell proliferation via B7-H4 and PD-L1 and increase Treg recruitment via CCL12. MIP-1β secreted by TAMs binds to CCR5, resulting in overexpression of P-selectin on the mesothelial cell surface. Cancer cells attached to P-selectin via CD24. TAM-derived soluble factors (such as FLT3L, leptin and HB-EGF) increase expression of MMP-9 which mediates spheroid spreading and invasion. TAM, tumor-associated macrophage; VEGF, vascular endothelial growth factor; KDR, kinase insert domain receptor; Flk, fetal liver kinase; UBR, ubiquitin protein ligase E3 component N-recognin; M-CSF, macrophage colony-stimulating factor; POSTN, periostin; CCL, chemokine (C-C motif) ligand; EGFR, epidermal growth factor receptor; CCR, C chemokine receptor; P-gp, P-glycoprotein; MRP, multidrug resistance-associated protein; SIRP, signal regulatory proteins; ASK, apoptosis signal-regulating kinase; ZEB, Zinc finger E-box binding homeobox; EMT, epithelial-mesenchymal transition; ICAM, intercellular adhesion molecule; VE-CAD, vascular endothelial-cadherin; B7-H4, B7 homolog 4; PD-L1, programmed cell death-ligand 1; MIP, macrophage Inflammatory Protein-1; Treg, regulatory T cells; FLT3L, FMS-like tyrosine kinase 3 ligand; HB, heparin-binding epidermal growth factor-like growth factor.

In ascites, the majority of immune cells are macrophages, which constitute >50% of MA cellular components (89). A study (17) showed that macrophages are present within all spheroids in ascites of 128 patients with OC at stage III. Compared with primary tumors, the amount of macrophages in spheroids is significantly increased and positively associated with proliferation while inversely associated with the prognosis of OC (17). In OC ascites, macrophages float in the peritoneal cavity or in the center of tumor spheroids. These macrophages may originate either from tissue-resident macrophages derived from the embryonic yolk sac or from infiltrating macrophages recruited from bone marrow-derived monocytes. They are induced to TAMs in the tumor microenvironment (TME), serving as an immunosuppressive cellular population, promoting tumor growth, immune escape, angiogenesis and metastasis. The role of TAMs in spheroid formation, survival and metastasis is summarized in Fig. 3.

### TAMs in angiogenesis and ascites formation

Increased permeability of vessels is a key pathophysiological process involved in ascites accumulation. TAMs are more likely to congregate at poorly vascularized sites (90) and promote angiogenesis in cancer by secreting VEGF and triggering revascularization (91). VEGF plays an essential role in angiogenesis and lymphangiogenesis by binding to receptors including kinase insert domain receptor (KDR)/fetal liver kinase (Flk)-1 and FMS-like tyrosine kinase (Flt)-1 (92). Han *et al* (93) found that TAM-derived chemokine CCL23 upregulates KDR/Flk-1 receptor expression in endothelial cells and promotes VEGF-mediated angiogenesis. VEGF levels are elevated significantly in OC ascites (94) and anti-VEGF treatment effectively suppressed tumor growth in a xenograft mouse model of OC and reduced ascites formation (95). Moreover, VEGF-A, VEGF-C and VEGF-D secreted by macrophages are involved in lymphangiogenesis dysfunction. Blockade of the VEGF-A/C/D pathway significantly inhibits the formation of chylous ascites in advanced OC mice (96). However, M2 macrophages downregulate the expression of very late antigen-4 (VLA4) when co-cultured with endothelial cells, decrease the levels of vascular cell adhesion molecule 1 (VCAM1) in endothelial cells and downregulate RAS-related C3 botulinum substrate 1 and reactive oxygen species, which resulted in decreased phosphorylation of proline-rich tyrosine kinase 2 and VE-cadherin. Therefore, M2 macrophages enhanced adhesion of endothelial cells and induced hypopermeability. Moreover, targeting the VLA4/VCAM1 axis enhances vascular integrity and eliminated the formation of ascites *in vivo* (97).

### TAM recruitment into ascites

MA in OC usually contain abundant TAMs, and the prognosis is poor (98). The mass recruitment of macrophages is associated with numerous factors, for example, chemokines CCL2 and macrophage colony-stimulating factor (M-CSF), which serve a key role in the recruitment of inflammatory monocytes to tumor sites and differentiation into TAMs. Blockade of CSF1R signaling, a key factor for macrophage recruitment, reduced the infiltration of macrophages, protected against vascular permeability by normalizing disorganized peritoneal vasculature and notably decreased ascites volume (99). Ubiquitin protein ligase E3 component n-recognin 5 (UBR5), is amplified and overexpressed in many types of cancers, particularly in OC (100). High expression of UBR5 in OC is associated with poor prognosis (101). High expression of UBR5 induces high expression levels of CCL12 and M-CSF, which recruit TAMs to facilitate spheroid formation. In mice, tumor cells with UBR5 overexpression are more invasive and lead to rapid death (18). Periostin (POSTN) produced by OC cells could enhance the recruitment of macrophages, which produced transforming growth factor β (TGF-β) to promote the production of POSTN by OC cells. Blocking this positive feedback pathway may decrease migration of macrophages to ascites (102). CD276 (B7-H3), a transmembrane protein, is an immune checkpoint member of the B7 family. Tumor-expressed CD276 contributes to macrophage recruitment in spheroids (103). Besides tumor cells attracting TAMs by releasing chemokines and cytokines, the TAMs themselves produce chemotactic mediators, such as CCL5, CXCL8, IL-1 receptor antagonist, CCL18, CXCL2 and CXCL3, which also contribute to monocyte/macrophage recruitment (104). Apoptosis signal-regulating kinase 1 (ASK1) belongs to MAP3K family, activating MAP2K-JNK/p38 cascades (105). Yin *et al* (106) found that ASK1 deficiency in vascular endothelium cells, but not TAMs, attenuated spheroid formation and peritoneal implantation in orthotopic OC mice. Mechanistically, ASK1 promoted macrophage transmigration via degradation of endothelial junction protein VE-cadherin. Pharmacological ASK1 inhibitor decreases tumor-induced vascular leakage, macrophage infiltration and tumor growth *in vivo* (105).

### TAMs polarization

Due to the plasticity of macrophages, undifferentiated macrophages (M0) can be polarized into two types, M1 and M2, which are distinguished by surface receptor expression, secretion pattern and function (107). The terms M1 and M2 were proposed by Mills *et al* (108) based on differences in arginine metabolism in macrophages from C57BL/6 and BALB/c mice, the effects of which are associated with differences between T helper (Th)1 and Th2 cell response. M1 macrophages produce a large number of pro-inflammatory cytokines under stimulation of Th1 cytokines such as IFN-γ and toll-like receptor (TLR) agonists such as lipopolysaccharide (LPS), and serve an essential role in anti-tumor response. M2 macrophages are stimulated by Th2 cytokines such as IL-4, IL-10 and TGF-β, promoting angiogenesis, and tissue repair (109). Due to the diversity of stimuli, M2 macrophages are further divided into M2a (IL-4 and IL-13), M2b (immune complex and LPS/IL-1), M2c (glucocorticoids, IL-10, TGF-β) and M2d (adenosine A2A receptor agonists and LPS) subtypes (109,110). Although the definition of M1-M2 macrophages provides a simplified paradigm for studying macrophage phenotype and function, this may oversimplify the complexity and diversity of macrophages, which exhibit mixed or unique phenotypes in many pathological conditions (111,112).

In the TME, polarized macrophages are known as TAMs. TAMs are composed of heterogeneous subpopulations, including M1 and M2 macrophages. TAMs predominantly express M2 macrophage markers and cytokines, such as CD206, CD163 and IL-10, and exhibit pro-tumor effects, and are therefore referred to as M2-like TAMs (113,114). By contrast, few TAMs in the TME express CD86 and CD80 markers and are referred to as M1-like TAMs, which typically exhibit anti-tumor effects (115,116). The ratio of M1/M2 is a prognostic indicator for OC. Patients with high M1/M2 ratio have a significantly longer overall and progression-free survival and platinum-free interval than patients with low M1/M2 (117). Plasticity is a key feature of macrophages. The phenotype of polarized M2-like TAMs can be reversed to M1-like TAMs to some extent (118). Therefore, shifting M2- to M1-like TAMs, rather than depleting TAMs, may serve as a treatment for cancer.

### TAMs promote spheroid formation

In ascites of OC, TAMs either float alone or are present in the center of spheroids encircled by cancer cells, primarily displaying M2-like phenotype with high expression of CD163 and CD206 (17). CD163^+^ TAM is rarely found outside the spheroids (119). TAMs participate in formation of spheroids, and are associated with prognosis of OC. CD68, a transmembrane glycoprotein that is widely expressed in monocytes, is considered a marker of TAMs in the TME (120). OC cases with high percentages of CD68^+^ TAMs (>14.5%) in spheroids have a significantly lower 5-year overall survival than those with low proportion (<14.5%) of CD68^+^ TAMs (17). In 3D co-culture system, CCL18/zinc finger E-box binding protein 1 (ZEB1)/M-CSF axis facilitates spheroid formation. During the spheroid formation stage, OC cells secretes M-CSF to induce TAMs to M2 polarization, and M2-like TAMs induce EMT of cancer cells, characterized by increased expression of mesenchymal markers (including ZEB1, SNAIL and TWIST) and decreased expression of E-cadherin. Mechanistically, TAMs release chemokine CCL18, which interacts with chemokine receptor 8 (CCR8) on the surface of OC cells, subsequently increasing the expression of ZEB1, a transcriptional factor which binds the promoter of M-CSF, and enhanced M-CSF expression. Overexpression of ZEB1 in OC cells promotes cancer cell-TAM spheroid formation *in vitro* and in mice (16). Since EMT is a key factor involved in invasion, metastasis and chemotherapy resistance (121), the CCL18/ZEB1/M-CSF feedback loop between OC cells and TAMs not only promoted formation of spheroids in ascites, but also led to faster and earlier transcoelomic metastasis of OC (16).

TAMs can secrete various cytokines which are essential for tumor cell proliferation and survival. EGF is one of these cytokines that form homodimers or heterodimers on the cell surface and mediate cell proliferation signal transduction. TAMs are key cellular sources of EGF secretion in tumor tissues (15). EGF/EGFR signaling between TAMs and cancer cells was essential for spheroid formation. Within large spheroids, TAMs displaying M2-polarization markers (including CD163, CD206 and arginase 1) are located in the center of spheroids and surrounded by EGFR^+^ cancer cells. TAM-derived EGF activated EGFR on cancer cells, then increases expression of VEGF-C and VEGFR3 in cancer cells. EGFR blockade with erlotinib may inhibit spheroid formation and transcoelomic metastasis *in vivo* (17). Moreover, EGF secreted by TAMs increases the expression of αMβ2 integrin in TAMs and ICAM-1 in cancer cells to facilitate adhesion between TAMs and cancer cells (17). Therefore, the EGF/EGFR/VEGF-C/VEGFR3/αMβ2/ICAM-1 signaling pathway serves a key role in OC progression (17).

### TAMs and anoikis resistance

Once floating in ascites, OC cells need to resist to anoikis, and multicellular spherical cells are more resistant to anoikis compared with individual cancer cells (60). TAMs serve a key role in anoikis resistance. Centrally located TAMs promote spheroid formation to provide a structural support OC cells to evade anoikis (17). STAT3 is an essential signal transduction molecule at the intersection of numerous pro-tumor signaling pathways (122), as well as mediating macrophage induction to M2-poralization (123). TAMs protected OC cells against anoikis via releasing several soluble factors, such as IL-6 and IL-10, which activated the STAT3 signaling in cancer cells, and promoted cell proliferation and peritoneal dissemination (123). When co-cultured with macrophages *in vitro*, especially M2 macrophages stimulated by M-CSF, ovarian cell line SKOV3 cells exhibit activation of the STAT3 signaling pathway (123). Hence, TAMs promote OC cell survival though enhanced anoikis resistance.

### TAMs and drug resistance

TAMs may promote spheroid resistance to chemotherapeutic drugs in ascites. In a 3D culture model of canine mammary gland tumor cell lines with or without macrophages, compared with homogeneous spheroids composed only of tumor cells, homogeneous spheroids composed of tumor cells and macrophages displayed increased cell viability when treated with doxorubicin. Compared with monolayer tumor cells, expression levels of VEGF, TGF-β, tumor necrosis factor-α-stimulated gene/protein-6 and drug resistance-related proteins such as P-glycoprotein and multidrug resistance-associated protein 1 are significantly increased in spheroids. Furthermore, doxorubicin-induced apoptosis and G2/M cell cycle arrest are decreased in the presence of tumor cells co-cultured with macrophages (69). CSCs are involved in tumorigenicity and drug resistance. TAMs regulated CSC activities by releasing milk-fat globule-epidermal growth factor-VIII, which activates STAT3 and Sonic hedgehog pathways in CSCs and contributes to their resistance to cisplatin *in vivo* (124). During the interaction between M2 macrophages and ovarian CSCs, paracrine Wnt is stimulated, which may enhance the aggressive phenotype of macrophages and cancer cells (125).

The presence of TAMs enhances drug resistance in OC spheroids, and TAMs can serve as a potential therapeutic target for treatment of OC. It is possible to reduce tumor cell resistance and improve cytotoxicity of drug therapy by blocking macrophage recruitment into the TME and resetting macrophage polarization (126). Recent studies revealed that blocking M2 macrophage polarization makes OC cells more sensitive to chemotherapeutic drugs including cisplatin and poly (ADP-ribose) polymerase inhibitors *in vitro* and *in vivo* (127-129).

### TAMs and immune escape

CD47-signal receptor protein-α (SIRPα) is the main innate immune checkpoint between macrophages and cancer cells. CD47 on the surface of tumor cells can release the signal 'don't eat me' by binding to the SIRPα receptor on the surface of macrophages, helping tumor cells to evade immune killing (130). Blocking the CD47/SIRPα signaling pathway can effectively promote phagocytosis of tumor cells by macrophages *in vitro* and *in vivo* (131). Blocking CD47 signaling with an oncolytic adenovirus carrying a SIRPα-IgG1Fc fusion gene (SG635-SF) significantly increases macrophage infiltration into the tumor and suppresses tumor growth in OC mice (132).

In the TME, TAMs have a markedly immunosuppressive effect on adaptive immune cells by releasing many cytokines, chemokines and enzymes, such as IL-10, TGF-β1, CCL12 and Arg-1. IL-10 causes naive CD4^+^ T cells to differentiate into Th2 cells, which suppress adaptive immunity, to allow malignant cells to escape immune surveillance (133). TGF-β1 may suppress T cell expansion through Smad3-dependent and -independent pathways (134). CCL22 secreted by TAMs establishes a chemokine gradient to induce regulatory T (Treg) cell migration into the local microenvironment, thereby increasing the proportion of Tregs (135). Tregs exist in ascites abundantly and accelerate tumor growth and progression via suppressing anti-tumor immunity. Macrophage-derived CCL23 induces CD8^+^ T cell exhaustion by upregulating molecules related to immune checkpoints, including cytotoxic T lymphocyte-associated antigen-4 (CTLA4), T cell immunoreceptor with immunoglobulin and immune receptor tyrosine-based inhibitory domain (TIGIT), T cell immunoglobulin and mucin domain 3 (TIM-3) and T cell immunoglobulin and mucin domain 3 (LAG-3) (136). TAMs express programmed cell death ligand 1 (PD-L1), PD-L2, CD80, and CD86, which restrict CD8^+^ T cell activation by binding to their receptors, PD1 and CTLA4 (137). B7-H3 not only serves as a costimulatory molecule that modulates T cell function directly (138), but also serves as an immune checkpoint involved in indirect T cell suppression via the CCL2-CCR2-M2 macrophage axis (139). B7-H4 is a recently identified B7 family molecule (140). A subpopulation of macrophages in OC ascites express B7-H4, a costimulatory molecule which decreases the proliferation and cytokine production of T cells (141).

### TAMs promote spheroid peritoneal dissemination

TAMs accelerate tumor metastasis by promoting expression of peritoneal mesothelial cell adhesion molecules and releasing growth factors and invasive proteases (142,143). During the metastasis of OC, P-selectin is overexpressed on the surface of mesothelial cells and cancer cells attach to P-selectin through CD24, resulting in increased adhesion between cancer and mesothelial cells (142). In a co-culture model with cancer and mesothelial cells and M2 macrophages, expression of P-selectin was regulated by M2 macrophages which secrete macrophage inflammatory pro1tein-1β (MIP-1β) that activates CCR5/PI3K signaling in mesothelial cells, resulting in upregulation of P-selectin on the mesothelial cell surface. MIP-1β treatment increases P-selectin expression in peritoneal mesothelial cells of mice and enhances OC cell adhesion *in vitro* and *in vivo* (142). Analysis of samples from patients with high-grade serous OC confirmed increased MIP-1β and P-selectin expression, suggesting that TAMs in ascites secrete MIP-1β, which increases expression of P-selectin on the surface of mesothelial cells (142).

OC spheroids depart from the primary tumor and reattach throughout the peritoneal cavity. Once cancer cells are implanted at new sites, formation of metastatic lesions is dependent on disintegration of spheroids and subsequent spread across the ECM. Soluble factors such as FMS-like tyrosine kinase 3 ligand, leptin, or heparin-binding EGF derived from TAMs are responsible for spheroids spreading across the underlying ECM. The common signaling pathway of these soluble factors is Janus kinase 2 (JAK2)/STAT3 activation followed by MMP-9 mediated spreading (144). In addition, macrophages increase the invasion ability of cancer cells though TNF-α and NF-κB pathways. Macrophages can release VEGF to promote the dissemination of OC cells onto the peritoneum of mice. In mice, deletion of macrophages alone results in decreased expression of VEGF, inhibition of ascites formation and peritoneal metastasis (143). Moreover, TAMs also secrete EGF to facilitate OC metastasis via activation of EGFR/ERK signaling and suppression of long non-coding (lnc)RNA inhibiting metastasis (LIMT) expression. Following co-cultured with M2 macrophages, OC cells showed greater migration capacity, and these effects could be reversed by inhibiting EGF and overexpressing lncRNA LIMT (145).

### TAMs as a target for treating OC

MA and spheroids are key for OC progression and recurrence, leading to relapse following classical therapy. TAMs play a critical role in spheroid formation and dissemination, as well as ascites formation. In addition, TAMs contribute to chemotherapeutic resistance and the suppression of the immune microenvironment characterized by enriched Tregs and exhausted CD8^+^ T cells, thereby promoting survival of cancer cells. Therefore, TAMs are a promising target in OC treatment. Strategies to target TAMs include blocking the recruitment of macrophages to the TME, shifting TAM polarization from M2 to M1 type, increasing phagocytosis of TAMs, blocking formation and intraperitoneal metastasis of TAM-tumor cell spheroids and improving chemotherapeutic sensitivity. Effects of TAM-based treatment for OC are summarized in Table I.

## 4. Conclusion

Cancer cells in OC ascites exist in two forms, individual cells and multicellular spheroids. Spheroids are divided into homospheroids and heterotypical spheroids, which contain tumor and non-tumor cells. The spheroids are involved in progression of OC, since they are more resistant to anoikis and chemotherapeutic drugs and are considered to be metastatic units. TAMs serve an essential role in OC progression. Recently, some groups demonstrated functions of TAMs in the formation of spheroids and dissemination (16,21,156). Although some researchers have revealed the discrete molecular mechanisms of spheroid formation, survival and metastasis (16,142), there are no data showing crosstalk between these pathways. Studies investigating the role of TAMs in OC spheroids typically use co-culture of TAMs and tumor cells in 3D culture model in the presence or absence of Matrigel or other scaffolds (16,157). Most studies have utilized OC cell lines and M2 macrophages to mimic OC cells and TAMs, respectively (16,142,144).These findings needed to be further clarified in patients. The majority of studies only co-cultured TAMs and tumor cells, and lack of other cellular components and non-cellular components present in the TME (16,144). These components are likely to influence TAM effects on the spheroids, making the results less convincing.

More molecules and signal pathways need to be investigated in spheroid formation, survival and metastasis, and the network among these molecules should be identified. Standardized methods are required for 3D culture model, such as description of size of spheroids, and presence or absence of scaffolds. Results obtained in primary cancer cells and TAMs from patients with OC are more persuasive than cell lines. Patient-derived organoids may mimic the TME. Cellular and non-cellular components in ascites can be separated from patients with OC and used to mimic the TME.

## Figures and Tables

**Table I:** TAMs as a therapeutic target in OC.

Mechanism	Model	Drug	Findings	Stage	First author, year	(Refs.)
Inhibition of TAM recruitment	Mouse	GW2580 (M-CSFR inhibitor)	GW2580, a M-CSF receptor kinase inhibitor, binds with M-CSFR on macrophages and monocytes, decreases the infiltration of M2 macrophages and significantly decreases the amount of ascites	Preclinical	Moughon, *et al*, 2015	(99)
	Cell (bone marrow-derived monocytes), mouse, human	Trabectedin	Trabectedin inhibits recruitment of circulating monocytes into tumor tissue and results in macrophage depletion by inhibiting production of CCL2, thus inhibiting OC progression	Clinical	Germano *et al*, 2013	(146)
	Human	Carlumab (CCL2 monoclonal antibody)	Carlumab is well-tolerated with evidence of transient free CCL2 suppression and preliminary antitumor activity. A patient with OC achieved CA125 reduction >50% and RECIST SD for 10.5 months	Ib clinical	Sandhu *et al*, 2013	(147)
	Human	Pexidartinib (M-CSFR tyrosine kinase inhibitor)	Combined with paclitaxel, 1/6 patients showed complete response (response duration 189 days) and 1/6 showed partial response (response duration 94 days)	I clinical	Wesolowski *et al*, 2019	(148)
Shifting TAM polarization from M2 to M1 type	Cell (murine epithelial ovarian cancer cell line ID8), mouse	Plerixafor (CXCR4 antagonist)	Plerixafor downregulates the expression of CXCL12 and CXCR4 and it promoted macrophage polarization from M2 to M1. Compared with αPD-1 therapy, plerixafor + αPD-1 significantly inhibits tumor growth and prolongs survival of tumor-bearing mice	Preclinical	Zeng *et al*, 2019	(149)
	Cell (murine RAW 264.7), mouse, human	Paclitaxel	Paclitaxel inhibits the polarization of M2 macrophages induced by the IL4/STAT6 pathway and reprograms them into M1 macrophages via the TLR4/NF-κB pathway, exerting an antitumor effect	Clinical	Wanderley *et al*, 2018	(150)
	Call (murine macrophage cell line Raw 264.7), mouse, HGSOC samples	Neferine	Neferine exerts antiangiogenic effects in HGSOC primarily by inhibiting polarization of M2 macrophages. Neferine decreases Arg-1 expression in macrophages, but increased the expression of iNOS, a marker of M1 macrophages	Preclinical	Zhang *et al*, 2018	(151)
	Cell (human acute monocytic leukemia THP-1), mouse	Infusion of IRF5 mRNA and IKKβ nanoparticles	Infusion of IRF5 mRNA and IKKβ nanoparticles reverses the immunosuppressive and tumor-supporting state of TAMs, significantly decreases the density of M2 macrophages and reprograms them into M1 macrophages and promotes the expression of IL-12, IFN-γ and TNF-α.	Preclinical	Zhang *et al*, 2019	(152)
	Cell (murine Raw 264.7), mouse	Chloroquine	Chloroquine serves as an immunomodulator and mediates its anti-tumor efficacy by increasing lysosomal pH to reset TAMs from M2 to M1 phenotype, and decreases immunosuppressive infiltration of myeloid-derived suppressor cells and Tregs, thus enhancing antitumor T-cell immunity	Preclinical	Chen *et al*, 2018	(153)
	Human	LPS	TAMs from ascites of patients with OC display. M2 phenotype. Upon TLR stimulation by LPS, TAMs acquire a classically activated functional phenotype (M1), release immunostimulatory cytokines (IL-12 and soluble IL-18) and efficiently trigger the cytolytic activity of NK cells	Clinical	Bellora *et al*, 2014	(154)
Increased phagocytosis of TAMs by immune checkpoint inhibition	Cell (human peripheral blood mononuclear cells), mouse, monkey	HuNb1-IgG4 (anti-CD47 nanobody)	HuNb1-IgG4 enhances macrophage-mediated OC cell phagocytosis. Because of its low affinity with red blood cells, it does not cause platelet aggregation or hemagglutination.	Preclinical	Ma *et al*, 2020	(155)
	Human	Hu5F9-G4 (CD47 antibody)	Of 13 patients with OC, two showed partial responses lasting 5.2-9.2 months	Clinical	Sikic *et al*, 2019	(131)
	Cell (human ovarian carcinoma SKOV3 and HO8910), mouse	SG635-SF (oncolytic adenovirus carrying SIRP-IgG1 Fc fusion gene)	SG635-SF blocks CD47 signaling in SK-OV3 and HO8910 OC cells expressing high levels of CD47. Macrophage infiltration into the tumor significantly increases and tumor cell killing is observed in xenograft tissue	Preclinical	Huang *et al*, 2020	(132)
Inhibition of spheroid formation	Mouse	Erlotinib (EGFR inhibitor)	Pharmacological blockade of EGFR or antibody neutralization of ICAM-1 in TAMs inhibits spheroid formation and OC progression	Preclinical	Yin *et al*, 2016	(17)
Inhibition of spheroid spreading on peritoneum	Mouse	Clodronate-containing liposomes	Clodronate-containing liposomes deplete peritoneal macrophages, which decreases tumor progression, as assessed by ascites formation and peritoneal metastasis. Inflammation facilitates ovarian tumor metastasis, primarily mediated by macrophages, which may involve stromal vascular endothelial growth factor production	Preclinical	Robinson-Smith *et al*, 2007	(143)
Improvement of chemotherapeutic sensitivity	Cell (human monocytes), mouse	Antisense oligonucleotide targeting circITGB6	circITGB6 stabilizes FGF9 RNA, which induces polarization of TAM toward the M2 phenotype, Antisense oligonucleotide targeting circITGB6 blocks M2 macrophage polarization and makes OC cells more sensitive to chemotherapeutic drug cisplatin	Preclinical	Li *et al*, 2022	(128)
	Cell (human ovarian cancer UWB1.289), mouse	STING agonists	STING agonists induce reprogramming of immunosuppressive myeloid cells, which inhibited TAM polarization toward M2 type and increases OC cell sensitivity to PARP inhibitors	Preclinical	Ding *et al*, 2023	(129)
	Cell (murine macrophage cell line RAW 264.7), mouse	Triptolide and miR-497	Exosome-liposome hybrid nanoparticle codelivery of triptolide and miR-497 promotes dephosphorylation of the overactivated PI3K/AKT/mTOR signaling pathway and increases ROS generation and polarization of macrophages from M2 to M1, making cells more sensitive to cisplatin *in vitro* and *in vivo*	Preclinical	Li *et al*, 2022	(127)

M-CSFR, macrophage colony-stimulating factor receptor; CCL2, Chemokine (C-C motif) ligand-2; CA125, carbohydrate antigen 125; RECIST, response evaluation criteria in solid tumors SD, stable disease; CXCR4, C-X-C chemokine receptor type 4; CXCL, C-X-C motif chemokine, αPD-1, anti-programmed death-1 antibody; TLR4, toll-like receptor 4, HGSOC, high-grade serous ovarian cancer; Arg-1, arginase-1; iNOS, inducible nitric oxide synthase; IRF5, interferon regulatory factor 5; IKKβ, IkappaB kinase-beta; TNF-α, tumor necrosis factor alpha; Tregs, regulatory T cells; TAMs, tumor-associated macrophages; LPS, lipopolysaccharide; NK cells, natural killer cells; SIRP, signal regulatory protein; EGFR, epidermal growth factor receptor; ICAM-1, intercellular adhesion molecule-1; circITGB6, circular RNA encoding integrin beta 6; FGF9, fibroblast growth factor 9; STING, stimulator of interferon genes; PARP, Poly ADP-ribose polymerase; miR-497, microRNA-497; PI3K, phosphoinositide 3-kinase; ROS, reactive oxygen species.

## Data Availability

Not applicable.

## Abbreviations

MA: malignant ascites
TAM: tumor-associated macrophage
ECM: extracellular matrix
EGFR: epidermal growth factor receptor
CAF: cancer-associated fibroblast
CSC: cancer stem cell
OC: ovarian cancer
M-CSF: macrophage-colony stimulating factor
UBR5: ubiquitin protein ligase E3 component n-recognin 5
EZH2: enhancer of zeste homolog 2
CAM: cancer-associated mesothelial cell
TME: tumor microenvironment
VEGF: vascular endothelial growth factor
VCAM1: vascular cell adhesion molecule 1
POSTN: periostin
ASK1: apoptosis signal-regulating kinase 1
TLR: toll-like receptor
